# Immediate Reconstruction of Complex Hand Trauma With Iliac Crest Bone Graft and 2 Pedicled Fasciocutaneous Skin Flaps: A Case Report

**Published:** 2010-03-10

**Authors:** Stéphane Stahl, Oliver Lotter, Adelana Santos Stahl, Hans-Eberhard Schaller, Nektarios Sinis

**Affiliations:** ^a^Department of Plastic, Hand and Reconstructive Surgery, Burn Center, BG-Trauma Center, Eberhard-Karl University of Tübingen, Germany; ^b^Department of Plastic and Reconstructive Surgery, Marienhospital, Stuttgart, Germany

## Abstract

**Objective:** The decision about primary or staged reconstruction of all structures in severe hand injuries is controversial. The purpose of this case report is to present a surgical protocol that lead to good functional results and rapid recovery after primary bone grafting with pedicled flap coverage of a complex hand injury. **Methods:** A case is reported in which 2 iliac crest bone grafts, an extended dorsal metacarpal artery flap and a heterodigital island flap, were performed at primary intervention to reconstruct the index and middle fingers of a 17-year-old patient. **Results:** Length and sensation of the digits were fully preserved while the destroyed joints were fused and covered with mobile soft tissue. Hereby, a satisfactory pinch grip and hand closure was restored, allowing the patient to return to work after only 2 months. **Conclusions:** Given proper planning and adequate debridement, primary reconstruction of all injured structures should be considered when dealing with complex hand injuries.

Good clinical judgment and methodical planning are mandatory to treat severe finger injuries. Since secondary interventions in reconstructed parts of the hand are the most challenging procedures in hand surgery, every attempt should be made to repair all structures immediately. Large soft-tissue defects of the fingers are a challenge to the reconstructive surgeon, especially when various digits are affected. The concept of early management of severe dorsal finger injuries to preserve functional length of the digits and provide adequate soft-tissue coverage is well established in the hand surgery literature.^1^^-^5 However, the case reported illustrates a unique management of large bone and dorsal soft-tissue defects of index and middle fingers combining two intrinsic hand flaps and bone grafting in an emergency 1-stage procedure with good functional outcome.

## CASE PRESENTATION

**Figure 1:**
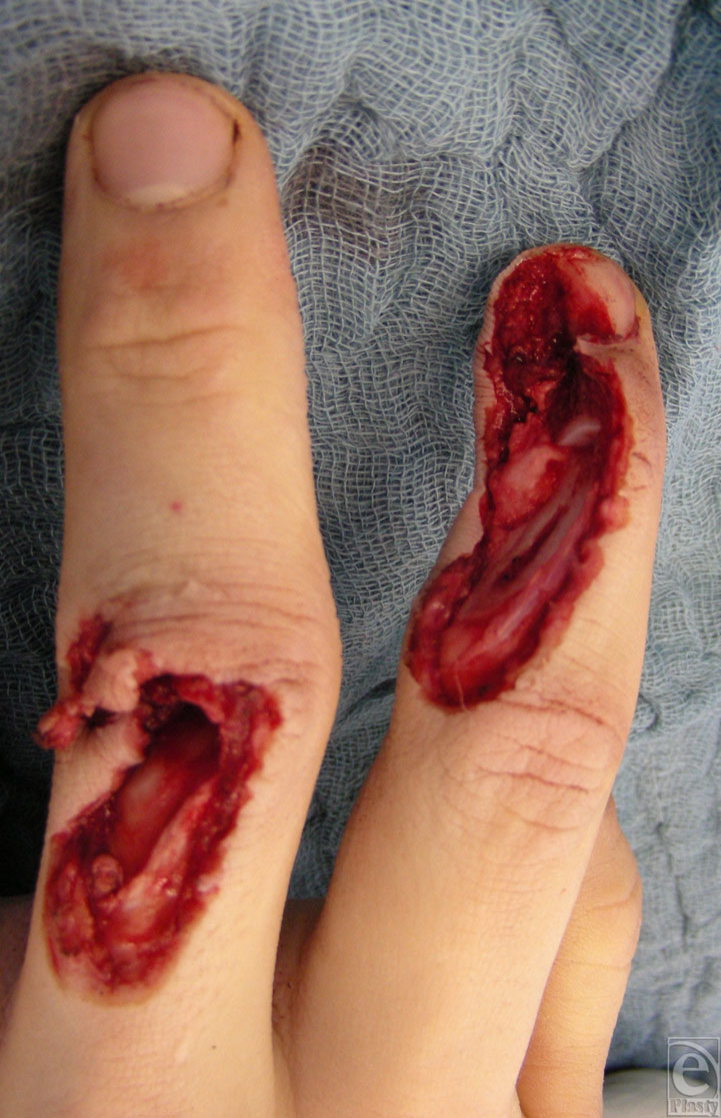
Defect of the ulnodorsal side of the index and middle fingers with exposed distal interphalangeal (DIP) II and proximal interphalangeal (PIP) III joints.

**Figure 2:**
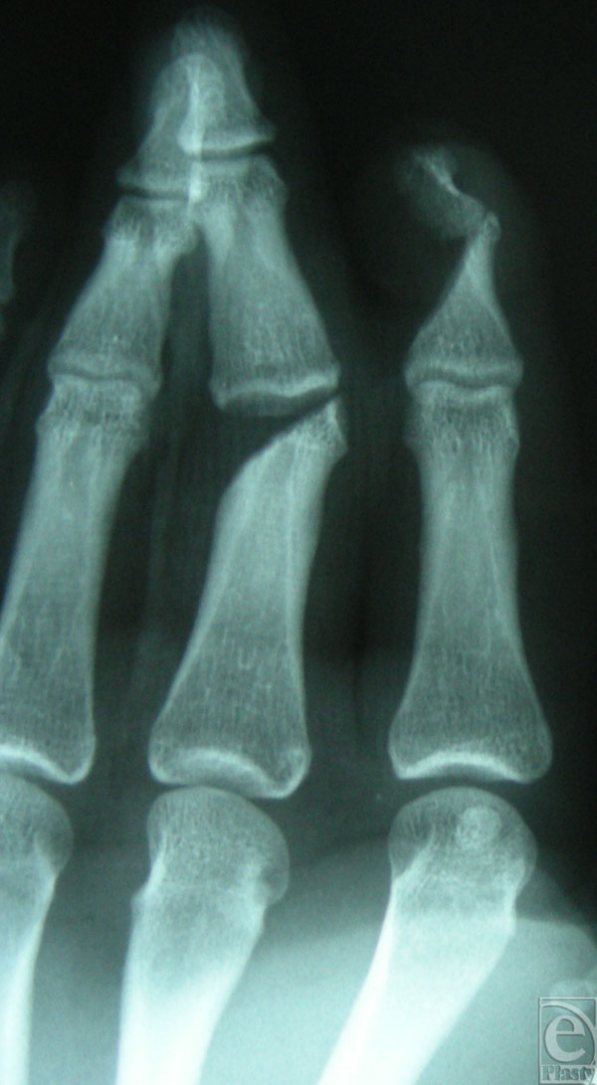
Anteroposterior radiograph showing the extension of articular destruction by the planer machine in the DIP II and PIP III joints.

A 17-year-old right-handed patient injured his left hand in a planer machine. Inspection of the traumatized hand revealed a severe injury on the dorsum of the index and middle fingers, with substantial soft-tissue defect. The index finger suffered complete destruction of the distal interphalangeal (DIP) joint, with a defect of two thirds of the extensor tendon and half of the nail bed and root. The middle finger suffered destruction of the proximal interphalangeal (PIP) joint and a defect of the ulnar lateral band (Fig 1). Both fingertips had preserved sensation and capillary refill. The anteroposterior radiograph shows the extent of the joint destruction of the middle phalanx of the third digit and the DIP joint of the index finger (Fig 2).

## OPERATIVE METHOD

Since the fingers' palmar arteries and nerves were fully preserved, amputation was rejected. First, an extended dorsal metacarpal artery (DMCA) flap was raised from the border of the retinaculum extensorum and transposed to the distal defect of the DIP joint of the index finger as described by Pelissier et al.^6^ Bone graft was harvested from the contralateral iliac crest for bone reconstruction and arthrodesis of the injured DIP joint of the index finger and PIP joint of the middle digit. The DMCA flap sufficed to cover the lateral nail fold after debridement of the nail bed. The ulnar lateral band of the middle finger was reconstructed with a tendon flap from the central slip of the arthrodesed PIP joint sutured to the lateral band. The heterodigital island flap was raised from the radial middle phalanx of the ring finger as described by Kojima et al.^7^ The donor defect was covered with full-thickness skin graft from the ipsilateral forearm. On postoperative day 10, physiotherapy was started, with splints to protect the arthrodesed joints. The patient was discharged from the hospital after 2 weeks. The inpatient care encompassed flap monitoring, an optimal wound-healing environment until individually molded orthoses were applied, and hand therapy was begun to ensure the best possible recovery and early return to work.

## FOLLOW-UP EVALUATION

**Figure 3:**
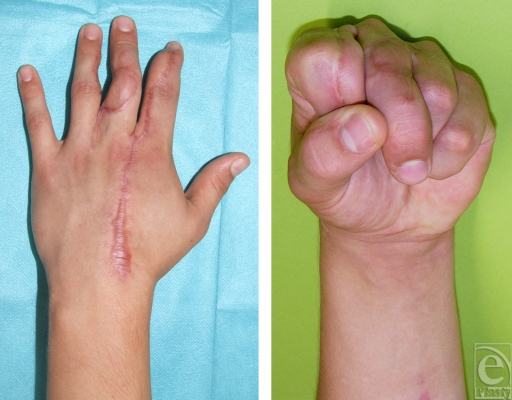
Postoperative functional result at 6 months. Note that a rotational deviation of the third digit remains after arthrodesis of the proximal interphalangeal joint.

**Figure 4:**
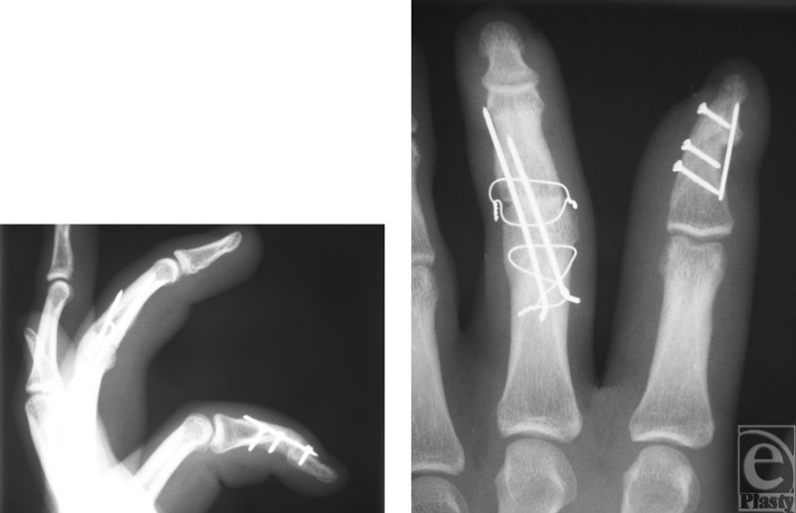
Postoperative radiograph at 6 months. Solidly consolidated arthrodesed joints after the interposition of iliac crest bone in the proximal interphalangeal joint of the middle digit and the distal interphalangeal joint of the index finger.

The patient returned to full-duty status 2 months after surgery. At follow-up examination after 6 months, a good functional result was noted (Fig 3) and the postoperative radiographs showed solid consolidation of the fused joints (Fig 4). The range of motion of all other joints was normal except a 30° extension deficit of the DIP joint of the index finger. Grip strength measurement with the JAMAR dynamometer at position 3 reached 30 kg on the left side and 50 kg on the right side. Key pinch strength averaged 10 kg on the left side and 13 kg on the right side. The patient was free of pain and assured he would request a reconstruction again. DASH (disabilities of the arm, shoulder, and hand) scored 26.66 points.

## DISCUSSION

Since secondary procedures are considerably hampered by the presence of scar tissue, primary reconstruction should be attempted, whenever possible. Early reconstruction following radical debridement reduces postoperative morbidity, infection rate, the number of procedures, and subsequent hospital stays.^8^ Exposed bone and joints dry out despite the best possible wound care, leading to necrosis and eventually infection.

The similar skin texture, color, and rapid execution make local flaps the best option in finger or thumb defects, whenever possible. Small free flaps such as from the snuff-box, thenar, first metacarpal artery (“kite” flap), first web space of the foot, dorsal middle phalangeal finger or the venous flaps are more time consuming and technically demanding.^9^ Hence, they should be reserved for the rare cases in which local reconstructive options are not available. Large free flaps such as fascia flaps or flaps from the forearm yield to syndactylization of the digits requiring another surgery for separation.

Sabapathy et al^5^ proposed the usage of lower abdominal pedicled flap and primary bone grafts for reconstruction of finger injuries with dorsal bone and soft-tissue defect, thus requiring syndactylization, immobilization of the upper extremity, and another procedure after 3 to 4 weeks. Although local pedicled flaps produce exposed donor site scars, they are our first choice for dorsal finger injuries because physiotherapy can commence within a few days. When repairing the extensor apparatus, gliding surfaces should be restored^10^ and early and sustained mobilization should be performed to lessen scar adhesion between the reconstructed bone and the extensor tendon.^11^ This is best achieved by definitive and functionally stable osteosynthesis.

The use of autogenic or allogenic bone graft in the primary reconstruction of open fractures of the hand or leg has not led to increased infection rates.^5^,^12^^-^15 Some authors advocate that before osseous reconstruction, soft-tissue defects have to be closed while segmental bone loss is temporally stabilized by external minifixators and antibiotic-loaded bone cement beads.^16^,^17^ Lister and Scheker^2^ achieved provisional stabilization by interposition of silicone blocks while performing a primary repair of tendons, nerves, vessels, and an emergency free flap. The present case illustrates that the functional benefits of primary repair of injured tendons and nerves also apply to early bone reconstruction leading to fast bony union, less immobilization, and rapid return to work.^3^,^16^,^18^^-^22 Edema, fibrotic changes, superficial infections as well as retraction of tendons, muscles and neurovascular bundles are various types of difficulties encountered in delayed microsurgical reconstruction.^23^ Godina^1^ indicated that early reconstruction decreases the number of reconstructive procedures needed, shortens recovery time, and reduces the physical and economic morbidity associated with the original trauma.

Proper planning of the surgery is decisive not only for good workflow among all team members but also for the adequate informed consent of the patient. Complex reconstructions require a thorough evaluation of the extent of the injury, the general condition of the patient, and of course an informed consent unless there is an immediate threat to the patient's life. If the extent of the injury cannot be appreciated before surgery, the procedure should be delayed until the patient can be informed about all options and consequences. The informed consent for reconstruction involving considerable donor site morbidity, such as primary toe transplants or free vascularized toe joint transfer, deserves particular discretion in the emergency setting since medical treatment might be considered as another potential source of damages if the patient seeks legal representation.

Only a realistic potential to improve function can justify grafting procedures and flap harvesting at the primary operation while the risks of infection and loss of grafts should be carefully weighed out. In complex reconstructions, the surgeon must not overlook the ultimate goal of useful function. To improve aesthetics of the presented results, a correctional osteotomy of the PIP joint of the middle finger along with the removal of osteosynthesis material and a minor scar correction could be considered during a second intervention. This case illustrates that immediate reconstruction including primary bone grafting can be performed safely as long as radical debridement and adequate soft-tissue coverage of bone are provided. Primary reconstruction allows early postoperative rehabilitation, leading to a good functional outcome and a rapid return to work.
